# Increased cellular immune responses and CD4+ T-cell proliferation correlate with reduced plasma viral load in SIV challenged recombinant simian varicella virus - simian immunodeficiency virus (rSVV-SIV) vaccinated rhesus macaques

**DOI:** 10.1186/1743-422X-9-160

**Published:** 2012-08-13

**Authors:** Bapi Pahar, Wayne L Gray, Kimberly Phelps, Elizabeth S Didier, Eileen deHaro, Preston A Marx, Vicki L Traina-Dorge

**Affiliations:** 1 Division of Comparative Pathology, Tulane National Primate Research Center, Tulane University School of Medicine, Covington, LA, USA; 2 University of Arkansas for Medical Sciences, Little Rock, AR, USA; 3 Departments of Chemistry and Physics, College of Sciences, Louisiana State University Shreveport, Shreveport, LA, USA; 4 Division of Microbiology, Tulane National Primate Research Center, Tulane University School of Medicine, Covington, LA USA; 5 Division of Microbiology, Tulane National Primate Research Center, 18703 Three Rivers Road, Covington, LA 70433, USA

**Keywords:** Simian varicella virus (SVV), Simian immunodeficiency virus (SIV), T-cells, Cytokine, Memory, Proliferation, Vaccine

## Abstract

**Background:**

An effective AIDS vaccine remains one of the highest priorities in HIV-research. Our recent study showed that vaccination of rhesus macaques with recombinant simian varicella virus (rSVV) vector – simian immunodeficiency virus (SIV) envelope and gag genes, induced neutralizing antibodies and cellular immune responses to SIV and also significantly reduced plasma viral loads following intravenous pathogenic challenge with SIV_MAC251_/CX1.

**Findings:**

The purpose of this study was to define cellular immunological correlates of protection in rSVV-SIV vaccinated and SIV challenged animals. Immunofluorescent staining and multifunctional assessment of SIV-specific T-cell responses were evaluated in both Experimental and Control vaccinated animal groups. Significant increases in the proliferating CD4+ T-cell population and polyfunctional T-cell responses were observed in all Experimental-vaccinated animals compared with the Control-vaccinated animals.

**Conclusions:**

Increased CD4+ T-cell proliferation was significantly and inversely correlated with plasma viral load. Increased SIV-specific polyfunctional cytokine responses and increased proliferation of CD4+ T-cell may be crucial to control plasma viral loads in vaccinated and SIV_MAC251_/CX1 challenged macaques.

## Findings

An effective human immunodeficiency virus type 1 (HIV-1) vaccine is needed to control the HIV pandemic. The use of live attenuated viruses as vaccines has demonstrated protection from rigorous homologous and heterologous viral challenges in macaques, thus providing critical proof-of-concept for the feasibility of the development of an effective HIV vaccine to prevent or limit HIV infection [1-3]. The result of clinical trial in Thailand testing with various subtype B canarypox-HIV-1 recombinant vaccine candidates and boosters containing subunit glycoprotein 120 or 160 did not generate strong cellular or detectable neutralizing antibody responses to HIV-1 yet showed marginally significant protection from infection [4]. A recent study with adenovirus/poxvirus and adenovirus/adenovirus-vector-based simian immunodeficiency virus (SIV) vaccine was shown to block acquisition of pathogenic heterologous, neutralization-resistant challenge virus in the rhesus macaque (RM) model [5]. A similar study with persistent cytomegalovirus (CMV) vector-based SIV vaccine was able to control highly pathogenic SIV infection by inducing effector memory T-cell responses [6]. So far, the development of an effective HIV vaccine that is capable of protecting new infection remains elusive.

Live-attenuated varicella-zoster virus (VZV) Oka vaccines have been shown to be safe and effective for immunization against VZV infection (chickenpox and shingles). This strongly immunogenic herpes virus vaccine backbone provides an attractive candidate for designing recombinant vaccines [7]. Periodic subclinical reactivation of VZV from latency may provide persistent immune re-stimulation to VZV and to foreign antigens. Due to stringent VZV host-range restrictions, the simian counterpart virus, simian varicella virus (SVV), with established RM models of both varicella and AIDS, provide an alternative experimental approach to investigate varicella pathogenesis and AIDS vaccine development [8,9]. Our recent study using recombinant SVV (rSVV) expressing SIV Gag + Env antigens (rSVV-SIV_MAC239_*Gag* and rSVV-SIV _MAC239_*Env*) demonstrated reduced plasma viral loads (VLs) in five immunized RMs (Experimental Group, EG) following intravenous SIV_MAC251_/CX1 challenge, when compared to four Controls vaccinated (rSVV-RSV_*G*_ and SVV-RSV_*M2*_) RMs (Control Group, CG) challenged with SIV_MAC251_/CX1 [10]. These results highlight the strengths and success of the SVV model to evaluate SIV/HIV vaccine candidates [10,11].

The present study investigates the role of cellular immune responses in rSVV-SIV immunized animals to define correlates of protection. All animals (EG and CG) were negative for HIV-2, SIV, type D retrovirus and STLV-1 infection at the beginning of the study. All the animals were housed at the Tulane National Primate Research Center (TNPRC) and under the full care of TNPRC veterinarians in accordance with the standards incorporated in the Guide to the Care and Use of Laboratory Animals (NIH) 78–23 (Revised, 1996). All veterinary procedures were performed only with sedated animals. All animal procedures were reviewed and approved by the Tulane Institutional Animal Care and Use Committee. The levels of T-cell proliferation, memory cell populations, polyfunctional T-cell responses in PBMC were assessed by flow cytometry and their responses were correlated with plasma VLs. In brief, T-cell immunophenotyping and Ki67 staining were performed using anti-CD3-FITC/PerCP (SP34-2), CD4-APC (L200), CD8-PE/PerCP (RPA-T8), CD95-FITC (DX2), CD28-PE (CD28.2) and anti-Ki67-PE (B56) monoclonal antibodies (mAbs) obtained from BD Biosciences (BD) as reported earlier [12]. Data was acquired on a FACS Calibur flow cytometer using BD CellQuest software and analyzed using FlowJo software, version 9.1. (TreeStar Inc., Ashland, OR).

**Figure 1:**
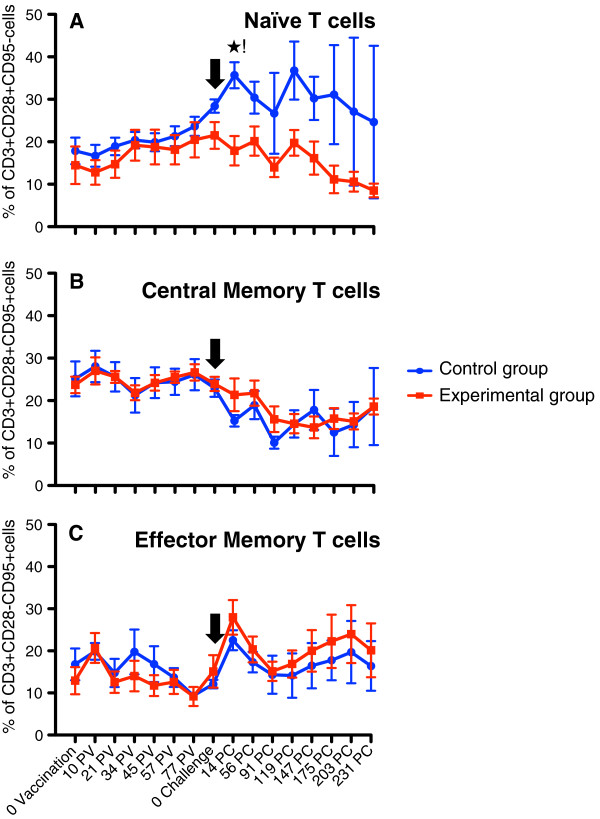
**Percentages of (A) naïve (CD28 + CD95-), (B) central memory (CD28 + CD95+) and (C) effector memory (CD28-CD95+) CD3+ T-cells in peripheral blood of both Experimental (EG) and Control group (CG) of macaques.** Note that naïve T-cell population showed progressive increase in both groups following immunization. A significant increase in naïve T-cell population was observed in animals from CG group compared to EG after SIV challenge. Following challenge there was increased and decreased expansion of effector and central memory T-cell population respectively in both EG and CGs of animals. Cells were gated through CD3^+^ “bright” T lymphocytes and at least 20,000 events were collected by gating on lymphocytes. Percentages of respective cell population represent cells out of total lymphocytes. PV and PC denote post vaccination and post challenge time points, in days, respectively. Black arrow shows the time of SIV_MAC251_ challenge. Asterisk (*) indicates a significant difference between EG (n = 5) and CG (n = 4) for the specified cell subset using the Student’s t-test, with a significance level of p < 0.05.

The naïve (CD28 + CD95-) T-cell population increased in both groups following rSVV immunization (Figure 1A), and is possibly due to heightened immune functioning, although neither increase was statistically significant. Following SIV challenge, however, the CG animals continued with a sharp rise from 28.4% on day of challenge (doc) to 35.2% on d14 post-challenge (d14pc) and remained high for the d231pc monitoring period (23%-37%). These findings were significantly different (p < 0.05) and in contrast to the homologous SIV challenged EG animals, with 20% naïve T-cells on doc and continued progressive decline to 8% at d231pc. This decline is likely due to a higher recruitment of naïve cells to SIV specific memory cells in this EG. Conversely, the central memory (CD28 + CD95+) T-cell population was maintained in both groups following immunization. After SIV challenge, a selective depletion of central memory T-cells was observed in both CG [22.9% (doc), 15.3% (d14pc), 10% (d119pc)] and EG [22% (d14pc), 15% (d119pc)] with no statistically significant differences within groups (Figure 1B). Finally, the effector memory (CD28-CD95+) T-cells peaked on d14pc with mean values 22.5% and 28.0%, in CGs and EGs respectively. This cell population decreased in both groups over the next two months to 15% followed by a gradual increase, slightly greater in the EG over the CG, although not statistically significant (Figure 1C). This sustained increase in the effector memory cell population following SIV challenge is thought to be due to the loss of the central memory T-cell population and induction of antiviral functions in plasma viral load reduction in both EG and CG animals.

**Figure 2:**
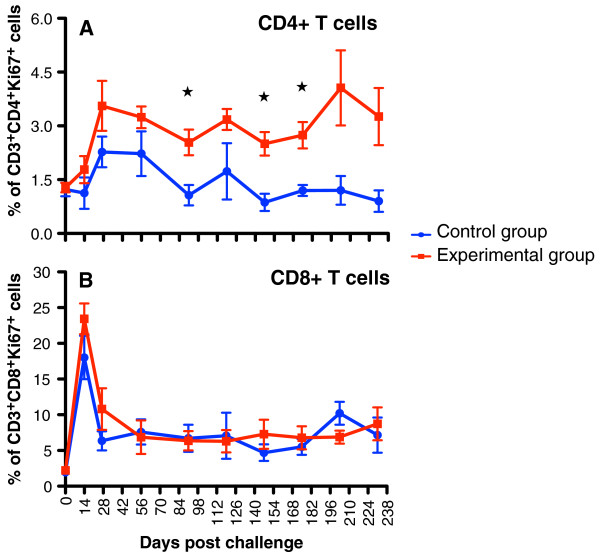
**Peripheral blood mononuclear cell proliferation assessed by %Ki67 expression is shown in CD3+CD4+ (A) and CD3+CD8+ (B) cells.** Both CD4+ and CD8+ T-cell proliferation increased following SIV_MAC251_ infection between 14 and 42 days of infection, but CD4+ T-cells in Experimental group (EG) maintained higher proliferation rates compared to Control group (CG). In contrast, proliferation in CD8+ T-cells both EGs and CGs returned to baseline thereafter. Cells were gated through CD3^+^ “bright” T lymphocytes and at least 20,000 events were collected by gating on lymphocytes. Asterisk (*) indicates significant differences between EG (n = 5) and CG (n = 4) for the specified cell subsets using the Student’s t-test, with a significance level of p < 0.05.

Next, we analyzed the level of proliferation in CD3+CD4+ T-cells and CD3+CD8+ T-cells to determine the impact of rSVV-SIV vaccination in response to SIV challenge (Figure 2). Following SIV challenge, an initial sharp increase in CD4+Ki67+ proliferating T-cells was observed in both vaccinated groups. The EG mean levels rose from 1.3% (doc) to 3.6% (d28pc) and maintained at significantly higher levels (P < 0.001) for the d231pc. The CG mean rose to 2.2% (d28pc), was transient however, decreased to baseline levels by d84pc and maintained lower than the EG for the remainder of the monitoring period. Similar increases in CD4+Ki67+ cells have also been observed in HIV-1 and pathogenic SIV infections [13-15]. An immediate increase in CD8+Ki67+ T-cells was observed in both EG and CG that was short-lived and returned to baseline by d28pc, supporting the similar expansion of CD8+ cells in peptide vaccine and SIV pathogenesis studies reported elsewhere [12,14].

**Figure 3:**
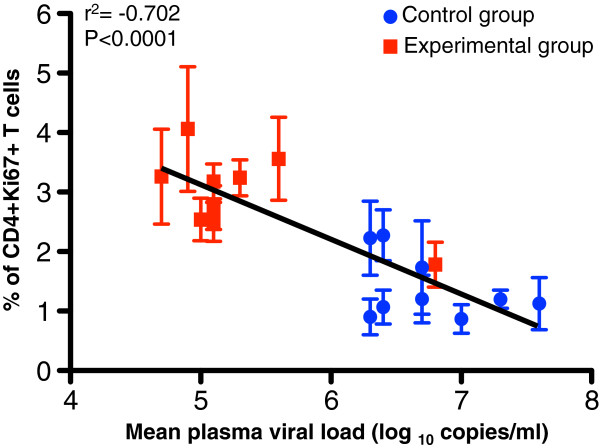
**An inverse correlation is shown between the CD3+CD4+ T-cell proliferation responses and plasma viral load in Experimental group (red squares, n = 5) and Control groups (blue circles, n = 4) of macaques following SIV**_**MAC251**_**challenge for all timepoints from d14 and d231.** Note that the lower viral loads demonstrated in the EG showed higher % of CD4+Ki67+ T-cells while the CG that had much higher viral loads had a lower % of CD4+Ki67+ T-cells. Interestingly, the one exception in EG overall trend was the high viral load at the d14 peak, yet the correlation held and at that point, the % of CD4+Ki67+ T-cells was low. Linear regression analysis of proliferating CD4+ T-cells and mean plasma viral load from all macaques was calculated using GraphPad Prism (version 5.0d, GraphPad software, CA).

Mean levels of plasma viremia were correlated with percentages of proliferating (Ki67+) CD4+ and CD8+ T-cells for both EG and CGs at each of the time points post SIV challenge (d14pc to d231pc). Pearson coefficient of determination analysis of these independent measures showed a highly significant inverse correlation of the proliferating CD4+Ki67+ T-cells and plasma viremia, regardless of treatment or timepoint (Figure 3). Linear regression analysis of these data resulted in a straight line with a slope 0.92 ± 0.15 (r^2^ = −0.702; p < 0.0001). All correlated values for the CG showed low levels of CD4+Ki67+ T-cells (0.9–2.2%) and high plasma VLs (log^6.3–7.6^ copies/ml) while all values but one for the EG had high levels of CD4+Ki67+ T-cells (2.5–4.1%) and low plasma viral loads (log^4.7 – 5.6^ copies/ml). The initial high viremic spike (log^6.8^ copies/ml) in the EG at d14pc also correlated with a low level of CD4+Ki67+ T-cells (1.8%) (Figure 3).

**Figure 4:**
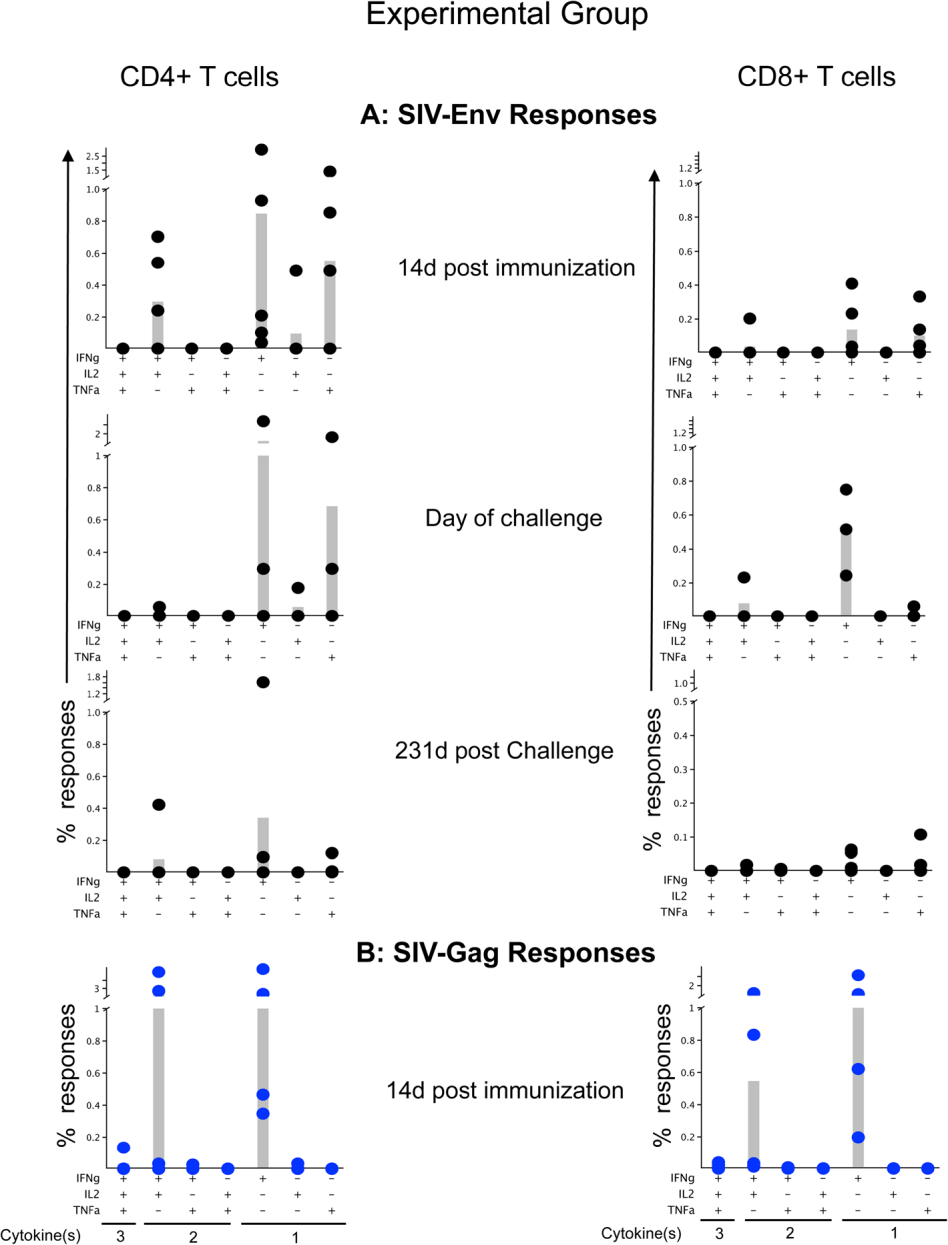
**Intracellular cytokine responses measured against SIV-Env (A) and SIV-Gag (B) antigens in Experimental vaccinated macaques were shown.** PBMC were unstimulated (medium control) or stimulated for 6 h with different SIV-Env and/or SIV-Gag peptide pools at 14d post immunization, day of challenge and 231d post challenge time points. Cells were gated on singlets, lymphocytes, followed by live cells and then on CD3+ T-cells and subsequently on CD3+CD4+ and CD3+CD8+ T-cell subsets. CD3+CD4+ or CD3+CD8+ T-cells were further analyzed for the presence of IFNγ, TNFα and /or IL2 positive cells by using Flowjo, and SPICE software. Increased polyfunctional responses were detected both in CD4 and CD8 T-cells, however the antigen specific CD4 responses were higher compared to CD8 specific responses. SIV-Gag specific responses were also higher compared to SIV-Env antigens. Individual animal responses are depicted by each dot and gray bars represent mean values of respective responses from all animals (n = 5). Positive symbols represent cells staining positive for a cytokine response, and minus symbols represent cells staining negative for a cytokine response. The presence of three different cytokine producing cells, two different cytokine producing cells and single cytokine producing cells are denoted under the bottom-most graphs (left to right) for each CD4 and CD8 cells as 3, 2 and 1 cytokine(s) respectively. The criterion for a positive cytokine response was a two-fold increase in frequency for that specific antigen and cytokine above the medium control culture. All values were subtracted from medium control before the analysis.

To assess the magnitude and functional characteristics of SIV specific CD4+ and CD8+ T-cells from both groups of animals, intracellular cytokine staining was performed with proper positive and negative controls as described earlier [16]. Resuspended PBMCs at 14 days post immunization (d14pi), doc and d231pc time points were stimulated with SIV-Gag (cat. 6204) and Env (cat. 6883) peptide pools (NIH AIDS Research & Reference Reagent Program) in the presence of 0.5ug/ml of anti-CD28 (clone CD28.2, BD) and anti-CD49d (clone 9F10, BD) mAbs. Following stimulation, the cells were stained for live/dead stain (Invitrogen), then surface staining with anti-CD3, anti-CD4, anti-CD8; and intracellular staining with anti-IL2 (MQ1-17H12), anti-IFNγ (4S.B3), and anti-TNFα (MAb11) mAbs. Data acquisition was performed on fixed stained cells using BD LSRII flow cytometer and analyzed using FlowJo software, version 9.1. [16] (Additional file 1: Figure S1). The analysis and presentation of cytokine expression was performed for each animal after proper antigenic stimulation and using SPICE software version 5.2, downloaded from http://exon.niaid.nih.gov/spice (Figure 4). In brief, cytokine responses produced by macaques are either monofunctional (mono, producing any one cytokine), bifunctional (bi, any combination of two different cytokines), or trifunctional (tri, producing three cytokines) cells and are diagrammatically represented in Figure 4. Recombinant SVV Experimentally immunized macaques (EG) generated SIV-Env specific monoCD4+, (mean values 0.50%, 0.66%, 0.12% on d14pi, doc, d231pc respectively) as well as biCD4+ (0.01%, 0.01%, 0.03% on d14pi, doc, d231pc respectively) (Figure 4, Additional file 2: Figure S2, Table 1). SIV-Env specific CD8+ responses were also demonstrated in the rSVV vaccinated EG macaques with monoCD8+ mean value responses of 0.08%, 0.18%, 0.02%; and biCD8+ mean values of 0.01%, 0.03%, 0.0% at d14pi, doc, d231pc respectively. In contrast, Control vaccinated (CG) macaques produced responses for SIV-Env specific cells with results of monoCD4 mean values of 0.0%, 0.0%, 0.19%; biCD4 mean values 0.0%, 0.0%, 0.0%; monoCD8 mean values 0.0%, 0.0%, 0.05%; and biCD8 mean values 0.0%, 0.0%, 0.0% on d14pi, doc, d231pc respectively (Figure 4, Additional file 2: Figure S2, Table 1). These data show that all EG macaques demonstrated substantial SIV specific cytokine responses in both CD4 and CD8 cells at pre challenge time points with CD4 responses greater than CD8 cytokine responses. All CG macaques were completely negative for SIV specific CD4+ and CD8+ cytokine responses at those prechallenge time points (Table 1). Postchallenge results showed positive cytokine expression in 4 out of 5 EG animals and 2 out of 4 CG animals (Additional file 2: Figure S2). Mean responses were lower in both groups (EG and CG), compared to the prechallenge responses, possibly due to immunosuppression in this late chronic phase of the SIV infection (Table 1).

**Table 1:** **Frequency of SIV antigen-specific cytokine producing cells in vaccinated animals**^**^**^

**Animal group**	**SIV Antigen**	Mono*	**d14pi**	**doc**	**d231pc**	Bi **	**d14pi**	**doc**	**d231pc**
**Experimental (EG)**	**Env**	**CD4**	0.50%	0.66%	0.12%	**CD4**	0.10%	0.01%	0.03%
	**Env**	**CD8**	0.08%	0.18%	0.02%	**CD8**	0.01%	0.03%	0.00%
	**Gag**	**CD4**	0.86%	ND	ND	**CD4**	0.79%	ND	ND
	**Gag**	**CD8**	0.43%	ND	ND	**CD8**	0.18%	ND	ND
**Control (CG)**	**Env**	**CD4**	0.00%	0.00%	0.19%	**CD4**	0.00%	0.00%	0.00%
	**Env**	**CD8**	0.00%	0.00%	0.05%	**CD8**	0.00%	0.00%	0.00%
	**Gag**	**CD4**	0.06%	0.00%	0.00%	**CD4**	0.05%	0.00%	0.00%
	**Gag**	**CD8**	0.05%	0.00%	0.00%	**CD8**	0.01%	0.00%	0.00%

(^) - Percentages are calculated means for each animal group.

Mono* - Monofunctional cytokine producing cells.

Bi** - Bifunctional cytokine producing cells.

ND – Not determined.

d14pi – d14 post-inoculation.

doc – day of challenge.

d231pc – d231 post-challenge.

Although we tested only one timepoint (d14pi) for SIV-Gag specific responses in EG, the values were 1.5-10 times higher than the SIV-Env specific responses at this d14pi time point. EG animals showed SIV-Gag specific monoCD4+ response mean of 0.86%, monoCD8+ mean of 0.43%, biCD4+ mean 0.79% and biCD8+ mean 0.18%, were higher when compared with CG responses for monoCD4+ mean 0.06%, biCD4+ mean 0.05%, monoCD8+ mean 0.05%, and biCD8+ mean 0.01% at d14pi. CG animals only were additionally tested for Gag-specific cytokine responses on doc and d231pc with no detectable responses for any animal in this group (Table 1). These findings demonstrate greater mono and polyfunctional responses during prechallenge time points in the EG over the CG animals and that CD4+ T-cells play a major role in inducing increased cytokine responses compared to CD8+ T-cells. In addition, although only at the one time-point tested, the gag-specific responses were greater than the Env-specific responses in EG animals compared to CG animals.

The increased CD4+ T-cell proliferation and profound SIV-Gag and Env specific cytokine responses in Experimental vaccinated macaques suggest that those proliferating CD4+ T-cells may be effector cells and their SIV-specific effector functions contributed significantly to control plasma VLs. Low to minimal neutralizing antibody responses in those rSVV-SIV vaccinated animals [10] also suggest that SIV-specific cytokine responses may play a crucial role in controlling plasma VLs and disease progression. Vaccine induced increased CD4+ T-cell proliferation and cytokine responses support earlier observations where HIV-specific CD4 T-cells were thought to be responsible for enhanced immunological control of HIV viremia either by helping CD8 T and B cells [17] or by direct antiviral effects [18,19]. Enhanced HIV-specific CD4 T-cells cytokine responses were also demonstrated in individuals that are able to control viral replication spontaneously in the absence of antiretroviral therapy [20,21]. Finally, with only a small percentage of HIV-specific CD4+ T-cells preferentially infected by HIV, the vast majority of uninfected CD4+ T-cells would be present and capable of inducing antiviral activity [22].

In conclusion, it is still unclear what constitutes the correlates of protection and what early immune responses are required to prevent early virus dissemination, viral replication, and viral transmission. In an effort to define those correlates, this vaccine study shows that increased CD4+ T-cell proliferation and increased SIV-antigen specific mono and polyfunctional CD4 and CD8 responses in the rSVV-SIV_Env/Gag_ vaccinated animals are key correlates of vaccine-mediated protection. These results show significant promise for rSVV-SIV vaccines as an effective preclinical approach to test potential recombinant AIDS vaccines with subsequent translation into rVZV-HIV vaccination in humans.

## Competing interests

The authors declare they have no competing interests.

## Authors’ contributions

BP, WLG and VTD conceived and designed the experiments; BP, KP, EH and VTD performed the experiments; BP, KP, ED, and VTD analyzed the data; BP, WLG, PM and VTD contributed reagents/materials/analysis tools; BP and VTD wrote the paper; All authors read and approved the manuscript.

## Supplementary Material

Additional file 1 **Figure S1.** Intracellular cytokine flow cytometry for IFNγ, TNFα and IL2 responses from a representative rSVV-SIVEnv and rSVV-SIVGag vaccinated rhesus macaque. Cells were gated first on singlets, lymphocytes, followed by live cells and then on CD3+ T-cells and subsequently on CD3+CD4+ and CD3+CD8+ T-cell subsets. The percentages of IFNγ, TNFα and /or IL-2 positive cells are shown in each upper box of each plot. Note that this vaccinated animal has an increased SIV-Gag specific IFNγ response. Click here for file

Additional file 2 **Figure S2.** Intracellular cytokine responses measured against SIV-Env and SIV-Gag antigens in Control vaccinated macaques were shown. PBMC were unstimulated (medium control) or stimulated for 6 h with different SIV-Env and/or SIV-Gag peptide pools at 14d post immunization (d14pi), day of challenge (doc) (**A & B**) and 231d post challenge (pc) time points (**C & D**). Cells were gated and analyzed as mentioned in Figure 4. All the animals had low to negative SIV antigen-specific responses detected at d14pi and doc of vaccination time points (**A & B**). Monofunctional responses were detected either in CD4 or CD8 T-cells from 2 out of 4 macaques and were limited to TNFα responses, however the antigen specific CD4 responses were higher compared to CD8 specific responses at d231pc time point (**C & D**). Individual animal responses are depicted by each dot and gray bars represent mean values of respective responses from all animals (n = 4). Positive symbols represent cells staining positive for a cytokine response, and minus symbols represent cells staining negative for a cytokine response. The presence of three different cytokine producing cells, two different cytokine producing cells and single cytokine producing cells are denoted under the bottom-most graphs (left to right) for each CD4 and CD8 cells as 3, 2 and 1 cytokine(s) respectively. The criterion for a positive cytokine response was a two-fold increase in frequency for that specific antigen and cytokine above the medium control culture. All values were subtracted from medium control before the analysis. Click here for file

## References

[B1] Pahar B Lackner AA Piatak M Jr Lifson JD Wang X Das A Ling B Montefiori DC Veazey RS Control of viremia and maintenance of intestinal CD4(+) memory T-cells in SHIV(162P3) infected macaques after pathogenic SIV(MAC251) challenge Virology 2009 387 273 284 19298994 10.1016/j.virol.2009.02.014 PMC2674129

[B2] Koff WC Johnson PR Watkins DI Burton DR Lifson JD Hasenkrug KJ McDermott AB Schultz A Zamb TJ Boyle R Desrosiers RC HIV vaccine design: insights from live attenuated SIV vaccines Nat Immunol 2006 7 19 23 16357854 10.1038/ni1296

[B3] Marthas ML Miller CJ Sutjipto S Higgins J Torten J Lohman BL Unger RE Ramos RA Kiyono H McGhee JR Efficacy of live-attenuated and whole-inactivated simian immunodeficiency virus vaccines against vaginal challenge with virulent SIV J Med Primatol 1992 21 99 107 1433273

[B4] Rerks-Ngarm S Pitisuttithum P Nitayaphan S Kaewkungwal J Chiu J Paris R Premsri N Namwat C de Souza M Adams E Vaccination with ALVAC and AIDSVAX to prevent HIV-1 infection in Thailand N Engl J Med 2009 361 2209 2220 19843557 10.1056/NEJMoa0908492

[B5] Barouch DH Liu J Li H Maxfield LF Abbink P Lynch DM Iampietro MJ Sanmiguel A Seaman MS Ferrari G Vaccine protection against acquisition of neutralization-resistant SIV challenges in rhesus monkeys Nature 2012 482 89 93 22217938 10.1038/nature10766 PMC3271177

[B6] Hansen SG Ford JC Lewis MS Ventura AB Hughes CM Coyne-Johnson L Whizin N Oswald K Shoemaker R Swanson T Profound early control of highly pathogenic SIV by an effector memory T-cell vaccine Nature 2011 473 523 527 21562493 10.1038/nature10003 PMC3102768

[B7] Arvin AM Mallory S Moffat JF Development of recombinant varicella-zoster virus vaccines Contrib Microbiol 1999 3 193 200 10599531 10.1159/000060324

[B8] Gray WL Simian varicella: a model for human varicella-zoster virus infections Rev Med Virol 2004 14 363 381 15386593 10.1002/rmv.437

[B9] Gray WL Simian varicella in old world monkeys Comp Med 2008 58 22 30 19793453 PMC2703154

[B10] Traina-Dorge V Pahar B Marx P Kissinger P Montefiori D Ou Y Gray WL Recombinant varicella vaccines induce neutralizing antibodies and cellular immune responses to SIV and reduce viral loads in immunized rhesus macaques Vaccine 2010 28 6483 6490 20654666 10.1016/j.vaccine.2010.07.018 PMC3061394

[B11] Ou Y Traina-Dorge V Davis KA Gray WL Recombinant simian varicella viruses induce immune responses to simian immunodeficiency virus (SIV) antigens in immunized vervet monkeys Virology 2007 364 291 300 17434552 10.1016/j.virol.2007.03.025 PMC1986657

[B12] Pahar B Cantu MA Zhao W Kuroda MJ Veazey RS Montefiori DC Clements JD Aye PP Lackner AA Lovgren-Bengtsson K Sestak K Single epitope mucosal vaccine delivered via immuno-stimulating complexes induces low level of immunity against simian-HIV Vaccine 2006 24 6839 6849 17050045 10.1016/j.vaccine.2006.06.050

[B13] Hazenberg MD Stuart JW Otto SA Borleffs JC Boucher CA de Boer RJ Miedema F Hamann D T-cell division in human immunodeficiency virus (HIV)-1 infection is mainly due to immune activation: a longitudinal analysis in patients before and during highly active antiretroviral therapy (HAART) Blood 2000 95 249 255 10607709

[B14] Kaur A Hale CL Ramanujan S Jain RK Johnson RP Differential dynamics of CD4(+) and CD8(+) T-lymphocyte proliferation and activation in acute simian immunodeficiency virus infection J Virol 2000 74 8413 8424 10954541 10.1128/jvi.74.18.8413-8424.2000 PMC116352

[B15] Giorgi JV Hultin LE McKeating JA Johnson TD Owens B Jacobson LP Shih R Lewis J Wiley DJ Phair JP Shorter survival in advanced human immunodeficiency virus type 1 infection is more closely associated with T lymphocyte activation than with plasma virus burden or virus chemokine coreceptor usage J Infect Dis 1999 179 859 870 10068581 10.1086/314660

[B16] Pahar B Wang X Dufour J Lackner AA Veazey RS Virus-specific T-cell responses in macaques acutely infected with SHIV(sf162p3) Virology 2007 363 36 47 17307212 10.1016/j.virol.2007.01.010 PMC1959567

[B17] Chevalier MF Julg B Pyo A Flanders M Ranasinghe S Soghoian DZ Kwon DS Rychert J Lian J Muller MI HIV-1-specific interleukin-21+ CD4+ T-cell responses contribute to durable viral control through the modulation of HIV-specific CD8+ T-cell function J Virol 2011 85 733 741 21047960 10.1128/JVI.02030-10 PMC3020027

[B18] Norris PJ Moffett HF Yang OO Kaufmann DE Clark MJ Addo MM Rosenberg ES Beyond help: direct effector functions of human immunodeficiency virus type 1-specific CD4(+) T-cells J Virol 2004 78 8844 8851 15280492 10.1128/JVI.78.16.8844-8851.2004 PMC479080

[B19] Soghoian DZ Streeck H Cytolytic CD4(+) T-cells in viral immunity Expert Rev Vaccines 2010 9 1453 1463 21105780 10.1586/erv.10.132 PMC3033049

[B20] Harari A Petitpierre S Vallelian F Pantaleo G Skewed representation of functionally distinct populations of virus-specific CD4 T-cells in HIV-1-infected subjects with progressive disease: changes after antiretroviral therapy Blood 2004 103 966 972 12958069 10.1182/blood-2003-04-1203

[B21] Rosenberg ES Billingsley JM Caliendo AM Boswell SL Sax PE Kalams SA Walker BD Vigorous HIV-1-specific CD4+ T-cell responses associated with control of viremia Science 1997 278 1447 1450 9367954 10.1126/science.278.5342.1447

[B22] Douek DC Brenchley JM Betts MR Ambrozak DR Hill BJ Okamoto Y Casazza JP Kuruppu J Kunstman K Wolinsky S HIV preferentially infects HIV-specific CD4+ T-cells Nature 2002 417 95 98 11986671 10.1038/417095a

