# Supplementary figures and images for: Increased cellular immune responses and CD4+ T-cell proliferation correlate with reduced plasma viral load in SIV challenged recombinant simian varicella virus - simian immunodeficiency virus (rSVV-SIV) vaccinated rhesus macaques
Source: Virol J. 2012 Aug 13;9:160. doi: 10.1186/1743-422X-9-160 (PMC3485174; doi:10.1186/1743-422X-9-160)

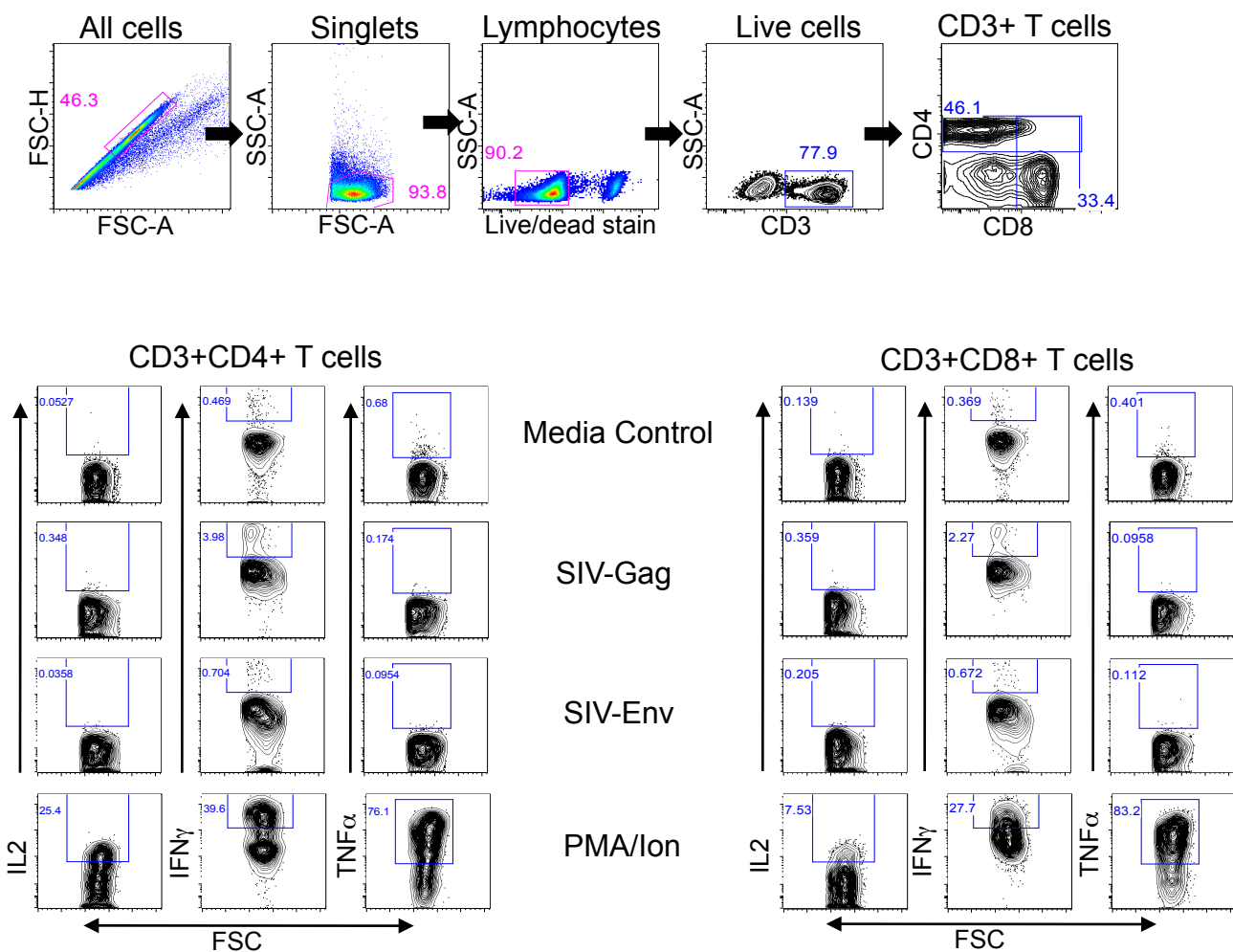

**Supplementary Figure S1**

Supplement: Additional file 1 — Figure S1. Intracellular cytokine flow cytometry for IFNγ, TNFα and IL2 responses from a representative rSVV-SIVEnv and rSVV-SIVGag vaccinated rhesus macaque. Cells were gated first on singlets, lymphocytes, followed by live cells and then on CD3+ T-cells and subsequently on CD3+CD4+ and CD3+CD8+ T-cell subsets. The percentages of IFNγ, TNFα and /or IL-2 positive cells are shown in each upper box of each plot. Note that this vaccinated animal has an increased SIV-Gag specific IFNγ response. [file 1743-422X-9-160-S1.pdf]

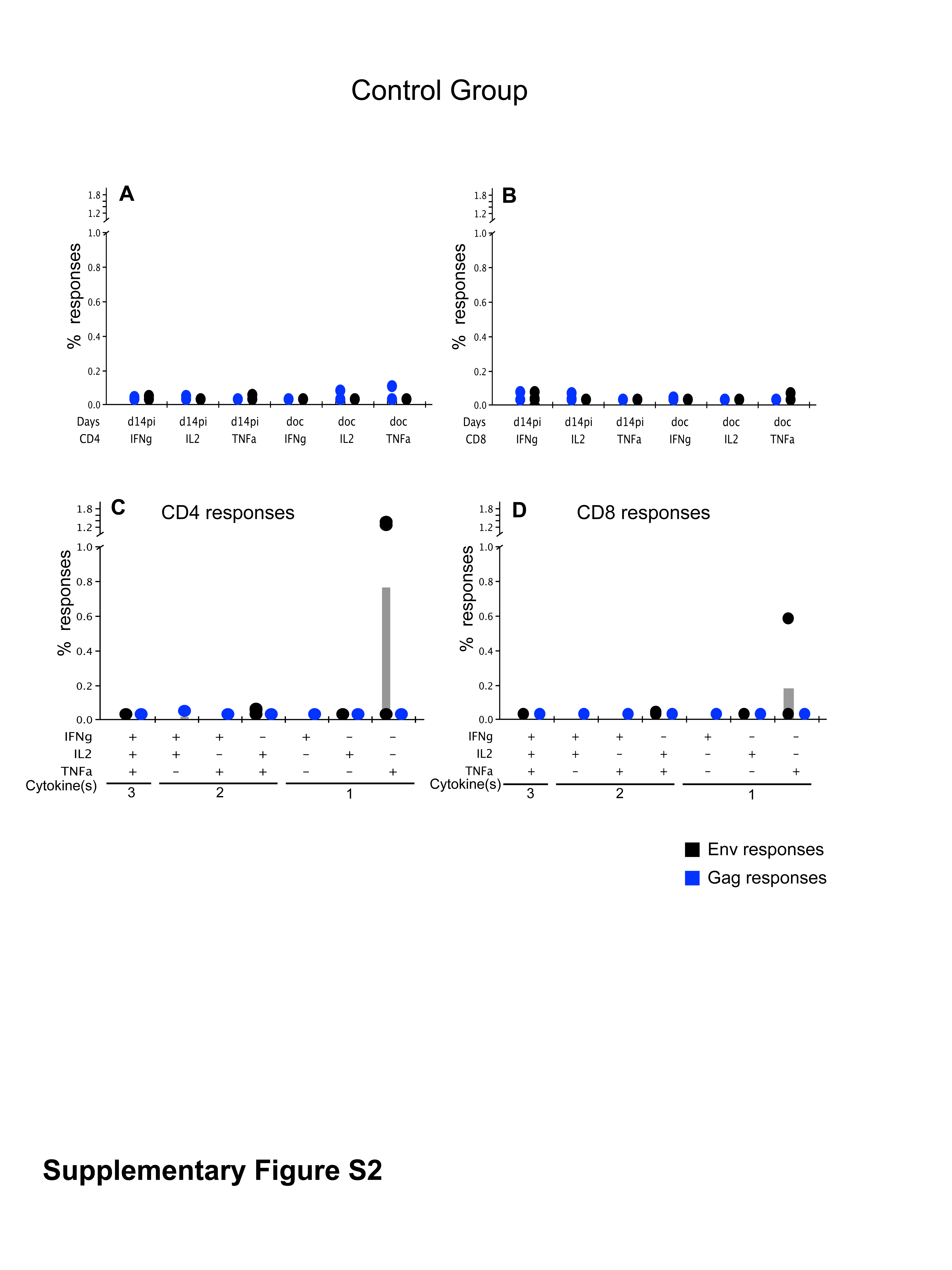

Supplement: Additional file 2 — Figure S2. Intracellular cytokine responses measured against SIV-Env and SIV-Gag antigens in Control vaccinated macaques were shown. PBMC were unstimulated (medium control) or stimulated for 6 h with different SIV-Env and/or SIV-Gag peptide pools at 14d post immunization (d14pi), day of challenge (doc) (A & B) and 231d post challenge (pc) time points (C & D). Cells were gated and analyzed as mentioned in Figure 4. All the animals had low to negative SIV antigen-specific responses detected at d14pi and doc of vaccination time points (A & B). Monofunctional responses were detected either in CD4 or CD8 T-cells from 2 out of 4 macaques and were limited to TNFα responses, however the antigen specific CD4 responses were higher compared to CD8 specific responses at d231pc time point (C & D). Individual animal responses are depicted by each dot and gray bars represent mean values of respective responses from all animals (n = 4). Positive symbols represent cells staining positive for a cytokine response, and minus symbols represent cells staining negative for a cytokine response. The presence of three different cytokine producing cells, two different cytokine producing cells and single cytokine producing cells are denoted under the bottom-most graphs (left to right) for each CD4 and CD8 cells as 3, 2 and 1 cytokine(s) respectively. The criterion for a positive cytokine response was a two-fold increase in frequency for that specific antigen and cytokine above the medium control culture. All values were subtracted from medium control before the analysis. [file 1743-422X-9-160-S2.tiff]
